# Radiation induces acute and subacute vascular regression in a three-dimensional microvasculature model

**DOI:** 10.3389/fonc.2023.1252014

**Published:** 2023-10-16

**Authors:** Dong-Hee Choi, Dongwoo Oh, Kyuhwan Na, Hyunho Kim, Dongjin Choi, Yong Hun Jung, Jinchul Ahn, Jaehoon Kim, Chun-Ho Kim, Seok Chung

**Affiliations:** ^1^ School of Mechanical Engineering, Korea University, Seoul, Republic of Korea; ^2^ R&D Research Center, Next&Bio Inc, Seoul, Republic of Korea; ^3^ Korea University-Korea institute of Science and Technology (KU-KIST) Graduate School of Converging Science and Technology, Korea University, Seoul, Republic of Korea; ^4^ Center for Systems Biology, Massachusetts General Hospital, Boston, MA, United States; ^5^ Laboratory of Tissue Engineering, Korea Institute of Radiological and Medical Sciences, Seoul, Republic of Korea; ^6^ George W. Woodruff School of Mechanical Engineering, Georgia Institute of Technology, Atlanta, GA, United States; ^7^ Center for Brain Technology, Brain Science Institute, Korea Institute of Science and Technology (KIST), Seoul, Republic of Korea

**Keywords:** radiation treatment, adverse effects, microvasculature-on-a-chip, radiation-injured vasculature, quantification

## Abstract

Radiation treatment is one of the most frequently used therapies in patients with cancer, employed in approximately half of all patients. However, the use of radiation therapy is limited by acute or chronic adverse effects and the failure to consider the tumor microenvironment. Blood vessels substantially contribute to radiation responses in both normal and tumor tissues. The present study employed a three-dimensional (3D) microvasculature-on-a-chip that mimics physiological blood vessels to determine the effect of radiation on blood vessels. This model represents radiation-induced pathophysiological effects on blood vessels in terms of cellular damage and structural and functional changes. DNA double-strand breaks (DSBs), apoptosis, and cell viability indicate cellular damage. Radiation-induced damage leads to a reduction in vascular structures, such as vascular area, branch length, branch number, junction number, and branch diameter; this phenomenon occurs in the mature vascular network and during neovascularization. Additionally, vasculature regression was demonstrated by staining the basement membrane and microfilaments. Radiation exposure could increase the blockage and permeability of the vascular network, indicating that radiation alters the function of blood vessels. Radiation suppressed blood vessel recovery and induced a loss of angiogenic ability, resulting in a network of irradiated vessels that failed to recover, deteriorating gradually. These findings demonstrate that this model is valuable for assessing radiation-induced vascular dysfunction and acute and chronic effects and can potentially improve radiotherapy efficiency.

## Introduction

Radiation treatment (RT) is a frequently employed anti-cancer treatment administered to nearly 50% of patients with cancer (1–3). Radiation is the process through which energy is transferred through waves that penetrate a range of materials, damaging tumor cell DNA directly or indirectly by reacting with bodily fluids to create reactive oxygen species (ROS) (4, 5). Apoptosis occurs when cellular DNA is damaged and is mediated via the tumor suppressor gene TP53 (4–7). Based on these fundamental principles, RT has been used to treat cancer but is well-known to induce diverse adverse effects, given its effects on both cancer and normal cells (8). Although innovative radiotherapy procedures such as linear energy transfer (LET), stereotactic radiosurgery (SRS), and stereotactic body radiation therapy (SBRT) have been developed, the intrinsic limits of radiotherapy persist (9, 10). Brain, breast, esophageal, head and neck, lung, and stomach cancers warrant a high rate of radiation therapy, and irradiation of these cancers impacts the brain, heart, and lungs. The stroma contains blood vessels that affect brain, heart, and lung functions. Cerebrovascular vessels deliver only essential substances to the brain to fulfill brain metabolism and protect the brain, whereas cardiovascular vessels regulate myocardial perfusion through vasoconstriction and vasodilation to meet the body’s metabolism. Pulmonary blood vessels prevent blood leakage, thereby facilitating gas exchange in the alveoli (8, 11–15).

Radiation induces both acute and chronic effects on blood vessels, including vascular depletion and inflammation. Vascular depletion is characterized by cellular pyknosis, increased vascular permeability, endothelial cell detachment from the basement membrane, disruption of the vascular structure, and decreased vascular density. Inflammatory alterations include an increase in intercellular adhesion molecule 1 and vascular cell adhesion molecule 1 adhesion molecules, the production of inflammatory cytokines, and the recruitment of inflammatory cells (1, 16). These modifications induce endothelial cell swelling, edema, and lymphocyte adherence and infiltration. Acute effects are predominantly mediated by radiation-induced apoptotic cell death, owing to DNA damage and ceramide production. DNA damage drives apoptosis by direct and indirect double-strand breaks (DSBs) and single-strand breaks. Conversely, the ceramide process occurs in a DNA damage-independent but membrane damage-dependent manner, mediated by the activation of acid sphingomyelinase (ASMase) and ceramide generation (17, 18). The produced ceramide then activates the MAPK8, mitochondrial, and death receptor pathways, which, in turn, activate caspase 1, 3, 6, and 9 and initiate apoptosis. These alterations in blood vessels gradually lead to pathological symptoms, including capillary collapse, atherosclerosis, endarteritis obliterans, telangiectasias, ischemia, and fibrosis (1, 18, 19).

Accordingly, these observations highlight the critical importance of examining vascular phenomena in response to radiation, given that this research could assist in overcoming the limitations of RT. Radiobiology research has historically employed animals or two-dimensional (2D) *in vitro* models. Animal models are frequently used in radiobiology research but are well associated with limitations, such as physiological differences between humans and animals, ethical issues, cost and time constraints, and challenges regarding high-throughput applications (20–22). Furthermore, mice have a lower capacity for genome maintenance than humans and a higher rate of somatic and germline mutations; therefore, they fail to accurately represent humans in studies that target DNA, such as radiotherapy (23, 24). Although 2D *in vitro* models are simple to set up and allow the use of high-throughput techniques, they are restricted by limitations, particularly the inability to replicate human physiological structures and functions (22, 25). By approximating the physiological structure and characteristics of human tissues and organs, microfluidic organ-on-a-chip has been key to overcoming these limitations (20, 21). On the basis of previous research, we have successfully constructed a 3D microvessel network model comprised of endothelial cells on a microfluidic chip and a radiation-injured vascular network model by irradiating it (26, 27). Radiation suppresses the formation of vascular networks, induces structural destruction and regression of the networks, and reduces the degree of perfusion. Moreover, vascular endothelial growth factor (VEGF) and sphingosine-1-phosphate (S1P) were incapable of repairing the radiation-induced damage. We demonstrated the structural degradation, functional impairment, and diminished recovery of microvessels caused by radiation from the perspectives of endothelial cells, endothelial cell layer, and vascular network.

## Materials and methods

### Fabrication and preparation of microfluidic chips

Microfluidic chips were fabricated via ultraviolet photolithography on a silicon wafer. On the patterned wafer, polydimethylsiloxane (PDMS) solution containing SYLGARD 184 silicone elastomer base and curing agent (weight ratio: 10:1, Dow Corning, USA) was cured for 2 h at 80°C. The reservoirs of the PDMS chip were punched with 4- and 1 mm biopsy punches. The punched chips were then sterilized twice at 120°C for 15 min each and dried in an 80°C oven for at least 6 h. After drying, sterile PDMS chips and cover glasses (Paul Marienfeld, Germany) were bonded using an oxygen plasma treatment (Femtoscience, Korea). After plasma treatment, the bonded chips were maintained in an 80°C oven for at least 24 h to recover the hydrophobicity of the microfluidic chip. The samples were then stored at room temperature until experimentation (28).

### Cell culture and cell seeding procedures in microfluidic chips

Human umbilical vein endothelial cells (HUVECs; Lonza Bioscience, Switzerland) and HUVEC CytoLight Green (GFP-HUVECs; Essen Bioscience, USA) were cultured in Endothelial Growth Medium 2 (EGM-2; Lonza Bioscience), and passages 6 to 8 were used for experiments. HUVECs were grown in a 75T flask until 80% confluence at 37°C and under 5% CO_2_ in a humidified incubator. Before cell seeding, a solution of 10 mg/ml fibrinogen (Sigma-Aldrich, US) in phosphate-buffered saline (PBS; Welgene, Korea) was prepared. Thrombin solution (50 U/ml, Sigma-Aldrich) was prepared in 0.1% bovine serum albumin (BSA; Sigma-Aldrich), while aprotinin solution (3 U/ml, Sigma-Aldrich) was prepared in distilled water. The fibrinogen-to-aprotinin ratio was 9:1 (29). HUVECs detached from the culture flask were centrifuged and suspended at a concentration of 8.4 × 10^6^ cells/ml. The cell suspension was then mixed with thrombin at a ratio of 49:1. The fibrinogen solution and cell suspension were mixed in a 1:1 ratio to yield 5 mg/ml fibrinogen and a cell suspension at 4.2 × 10^6^ cells/ml. A mixture of these two substances was injected into the gel channel. The gel-filled chip was then incubated for 10 min at 37°C in a 5% CO_2_ incubator to induce the gelation of the fibrin gel. After 10 min, all media channels were filled with EGM-2 containing 20 ng/ml of vascular endothelial growth factor – A165 (VEGF165; Peprotech, USA). As previously described, HUVECs were suspended at a concentration of 7 × 10^6^ cells/ml and mixed with fibrin gel for recovery experiments. Subsequently, the mixture was injected into a gel channel. PBS was mixed with thrombin at a ratio of 49:1. The fibrinogen and PBS solutions were mixed in a 1:1 ratio, then injected into the second gel channel. EGM-2 containing 20 ng/ml VEGF165 was injected into the medium channel adjacent to the gel channel containing HUVECs, while EGM-2 containing 100 ng/ml VEGF165 and 500 nM sphingosine-1-phosphate (S1P; Sigma-Aldrich) were injected into the medium channel adjacent to the avascular gel channel. The media in the chips was refreshed daily.

### Irradiation

Briefly, cells were exposed to γ-rays from a ^137^Cs irradiation source (Biobeam 8000; Gamma-Service Medical GmbH, Germany). In one instance, cells were irradiated 12 h after cell seeding to determine the effect of radiation on neovascularization. In another instance, four days after cell seeding, cells were irradiated to identify the effect of radiation on the mature vascular network. Additionally, cells were irradiated three days after cell seeding to confirm the recovery potential of the irradiated vasculature. Cells were exposed to radiation doses of 4, 8, and 16 Gy, with unirradiated cells serving as controls.

### Immunostaining

The vasculature models were fixed with 4% paraformaldehyde for 30 min and permeabilized with 0.1% or 0.5% Triton X-100 for 15 min at room temperature. The membranes were blocked at room temperature for 1 h using 5% BSA to reduce non-specific binding. The chips were then incubated at 4°C for 24 h with primary antibody solutions against anti-VE cadherin (Abcam, UK), anti-Laminin (Abcam), anti-CD31 (Abcam), anti-ZO-1 (Invitrogen, US), or anti-γH2AX (Abcam). After incubation, the chips were twice washed with PBS. Subsequently, a secondary antibody solution containing Alexa Fluor® 488 (Molecular Probes, US), 4′,6-diamidino-2-phenylindole (DAPI, Sigma-Aldrich), or rhodamine-phalloidin (Molecular Probes) was injected into media channels, and the chips were maintained at room temperature for 1 h. Subsequently, the cells were washed again, and confocal laser scanning microscopy images were captured (LSM700, Carl Zeiss, Germany).

### Viability and apoptosis of irradiated blood vessels

The endothelial cell viability was measured using Hoechst33342 (Molecular Probes), calcein-AM (Invitrogen), and ethidium homodimer-1 (Invitrogen). Briefly, 100 μl EGM-2 containing 1 drop/ml Hoechst33342, 2 μM calcein-AM, and 2 μM ethidium homodimer-1 was injected into the chip and incubated for 30-60 min. After incubation, the chip was washed with PBS and imaged using fluorescence microscopy. Irradiated endothelial cells were evaluated for apoptosis using NucView488 Caspase-3 (Biotium, US) and Hoechst33342. The chip was incubated with 100 μl EGM-2 containing 5 μM NucView488 and 1 drop/ml Hoechst33342 for 30 to 60 min. Following incubation, the chip was washed with PBS and examined.

### Reverse transcription-quantitative PCR

After 6 or 24 hours of radiation exposure, the fibrin gel was treated with TrypLE Express (Gibco, US) at 37°C for 2 hours to extract endothelial cells. For one group, endothelial cells were harvested from over twenty chips, and approximately 900 ng of total RNA was extracted using the RNeasy Plus Mini Kit (Qiagen, Germany). Using a Nanodrop Spectrophotometer (ND-1000, Thermo Fisher Scientific, US), the purity and concentration of extracted RNA were determined. RNA was reverse transcribed to complementary DNA using a high-capacity RNA-to-cDNA kit (Applied Biosystems, US). RT-qPCR was carried out using cDNA, endothelial cell-specific primers, proliferation- and apoptosis-related primers listed in **Supplementary Table 1**, and QuantiTect SYBR Green PCR Kits (Qiagen). PCR was conducted using a StepOne Real-Time PCR System (Applied Biosystems). GAPDH was used as the control to normalize experimental samples (30).

### Vasculature structure analysis

Blood vessel structures were analyzed using fluorescence images. The contrast of raw images was enhanced, and image thresholding was performed using the ImageJ software (National Institutes of Health, Bethesda, MD). To decrease false positive measurements, 5-unit Gaussian blur filter was applied to the threshold images. After filtering, the images were skeletonized for the vessel structure analysis (**Figure S1**). ImageJ software was used to measure branch length, branch number, junction number, vasculature area, and sprouting length (26).

### Permeability and blockage of the vasculature

On day 7, 60 μl of 10 μM FITC-dextran (70 kDa; Sigma-Aldrich) was injected into the medium channel to determine blood vessel permeability. After perfusing the vessel with dextran solution using hydrostatic pressure, the pressure was removed to eliminate convection. Fluorescent images were captured every 10 s for 90 s. Assuming that the blood vessel had a circular cross-section, the permeability coefficient was determined as follows:


$$P_{d}=\frac{1}{I_{1}-I_{b}}\left(\frac{I_{2}-I_{1}}{\Delta t}\right)\frac{d}{4}$$

where I_1_ is the average initial intensity in the measuring window, I_b_ is the background fluorescence intensity, I_2_ is the average intensity after *t* s, and d is the vessel diameter (31). Fluorescent images were analyzed using the ImageJ software.

To measure vascular blockage, endothelial cells were stained with Hoechst33342 according to the method described for the viability and apoptosis of vessel sections. After Hoechst33342 staining, 60 μl of 10 μM 70 kDa FITC-dextran was injected into one of the medium channels. Fluorescent images were captured 30 s after introducing dextran into the channel. After imaging, the chips were washed thrice with PBS and fixed with 4% paraformaldehyde. The cells were stained with rhodamine-phalloidin, as described in the immunostaining section. The blocked blood vessels were quantified by comparing the vessel area perfused with dextran to the vessel area stained with F-actin.

### Statistics

The statistical calculations of the results were performed by Prism (GraphPad Software Inc., US) and data were expressed as mean ± standard deviation (SD) with at least three biological replicates or as mean ± standard error (SEM) with at least three technical replicates. The significance of the data between the two groups was determined using unpaired, two-tailed Student’s t-tests. A *p*-value of less than 0.05 was considered statistically significant: *p< 0.05, **p< 0.01, ***p< 0.001, ****p< 0.0001.

## Results

### Development of 3D vasculature on a microfluidic chip

**Figure 1:**
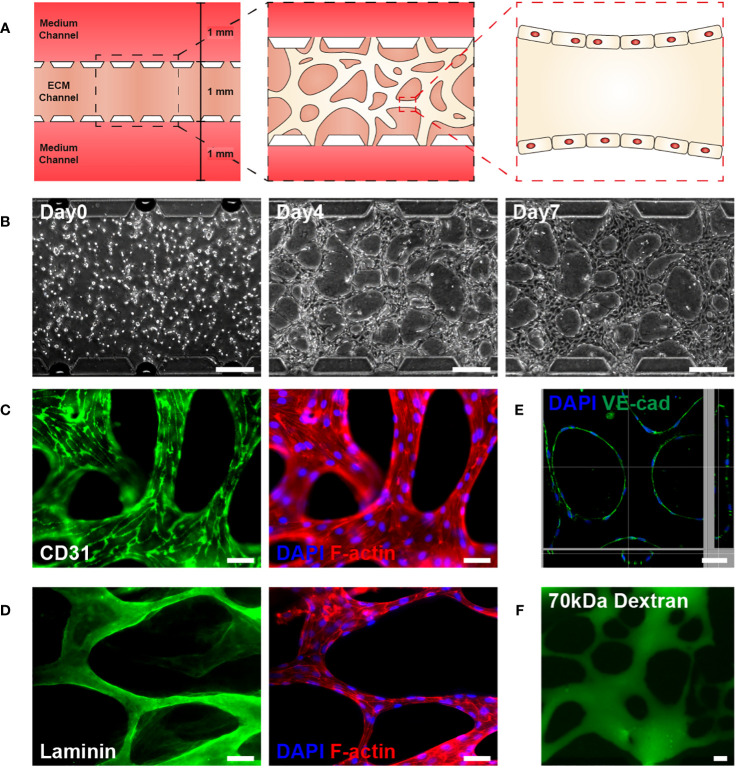
Development of a three-dimensional (3D) microvasculature on a microfluidic chip. **(A)** Schematic of 3D microvasculature-on-a-chip. The ECM is filled in the middle channel of the chip, and the medium is supplied on both sides of the channel. The 3D vasculature is formed in the ECM channel, along with the formation of the blood vessel monolayer, as shown in the schematic. The height and length of channels are 180 µm and 5.1 mm. **(B)** Phase contrast images illustrating the progression of the vasculature’s morphology over time (Day 0, 4, 7). Scale bar, 200 μm. **(C)** Fluorescent images of CD31 (green, mature endothelial cells), DAPI (blue, DNA in nuclei), and phalloidin (red, F-actin) in the vasculature. Scale bar, 50 μm. **(D)** Fluorescent images of the vasculature showing laminin (green, basement membrane), DAPI (blue), and phalloidin (red). Scale bars, 50 μm. **(E)** A 3D z-stack confocal image of vasculature illustrating the lumen structure of vasculature. Co-staining with DAPI and VE-cadherin, an endothelial adherens junction marker, was performed. Scale bar, 50 μm. **(F)** Perfusion of vasculature with 70 kDa FITC-dextran. Fluorescent and perfusion images were taken on day 7. Scale bar, 50 μm. ECM, extracellular matrix.

The microfluidic chip displayed a gel channel and two medium channels. HUVECs and fibrin gel were injected into the center gel channel, while EGM-2 medium containing 20 ng/ml VEGF165 was injected into the two medium channels (**Figure 1A**). HUVECs, injected as single cells, could form a 3D vascular network in the fibrin extracellular matrix (ECM) within 4 days with the aid of VEGF and EGM-2, which was remodeled via pruning and intussusception (**Figure 1B**) (32, 33). Co-staining with CD31, a marker for mature endothelial cells, DAPI, and F-actin confirmed that the network was a 3D vasculature, with HUVECs aligning tube-like structures (**Figure 1C**) (34). During vasculature formation, HUVECs reportedly produce a basement membrane on the basal surface of the endothelial monolayer (33, 35). Prior to HUVEC seeding in fibrin gel, laminin, a major component of the basement membrane near the vasculature, was absent. However, after HUVEC seeding in fibrin gel, laminin was detected near the vasculature (**Figure 1D**). The stabilized 3D vasculature presented a lumen structure for material transport, one of the primary functions of blood vessels (**Figure 1E**), opening toward the medium channels to permit perfusion of 70 kDa dextran into the vasculature (**Figure 1F**).

### Radiation disrupts new blood vessel formation

**Figure 2:**
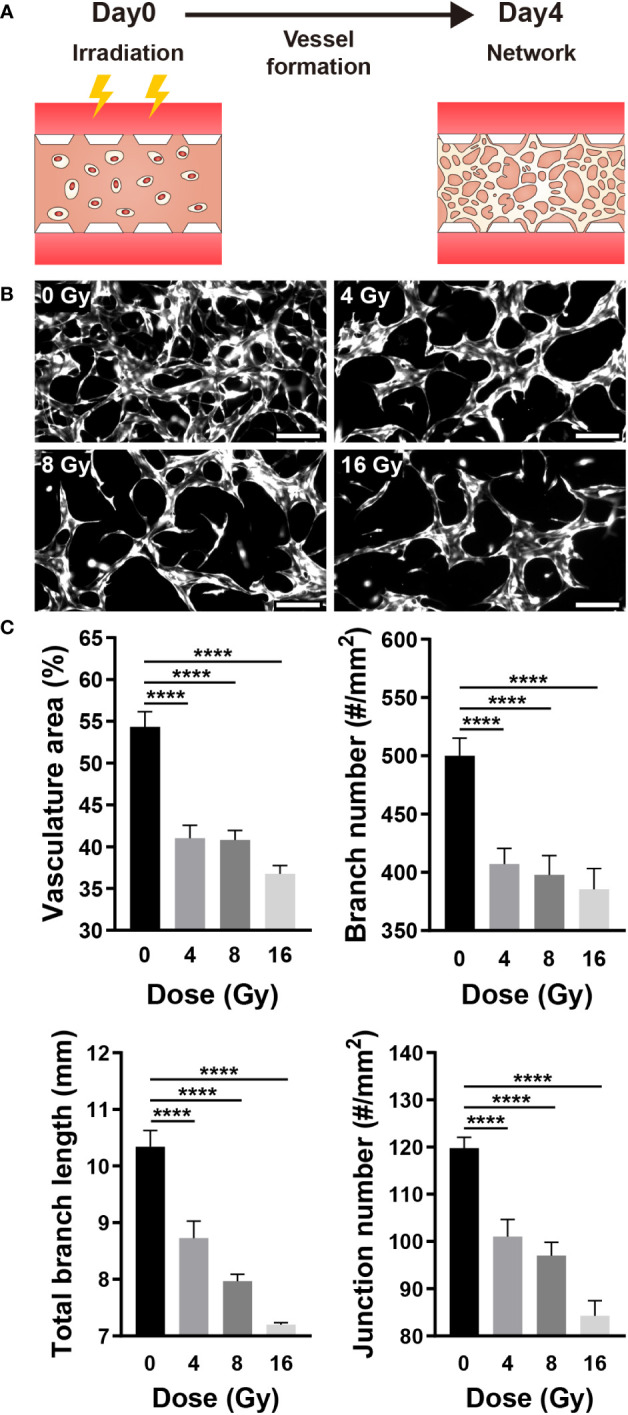
Radiation disrupts neovascularization. **(A)** Schematic illustrating experimental conditions for analyzing the effect of radiation on neovascularization. **(B)** Control and irradiated vascular networks on day 4. Scale bars, 200 μm. **(C)** Quantitative indices such as vasculature area, number of branches, length of total branches, and number of junctions were measured to analyze the vascular structure of control and irradiated vessels (mean ± SD, n = 9). ****p< 0.0001. SD, standard deviation.

Vasculogenesis is the formation of new blood vessels via the differentiation, migration, and fusion of endothelial cells and progenitors (32). When formed blood vessels are exposed to proangiogenic factors such as VEGFA, endothelial cells chemotactically migrate and proliferate toward proangiogenic factors to form a new blood vessel, a process referred to as angiogenesis (33). To determine the effect of radiation on neovascularization, HUVECs were irradiated 12 h after injection into a gel channel with fibrin gel, and vascular formation was analyzed for 4 days (**Figure 2A**). The 3D vasculature formed by irradiated HUVECs exhibited a simple structure with few branches (**Figure 2B**). Furthermore, the area, overall branch length, branch number, and junction number of the vasculature formed by irradiated HUVECs decreased dose-dependently (**Figure 2C**).

**Figure 3:**
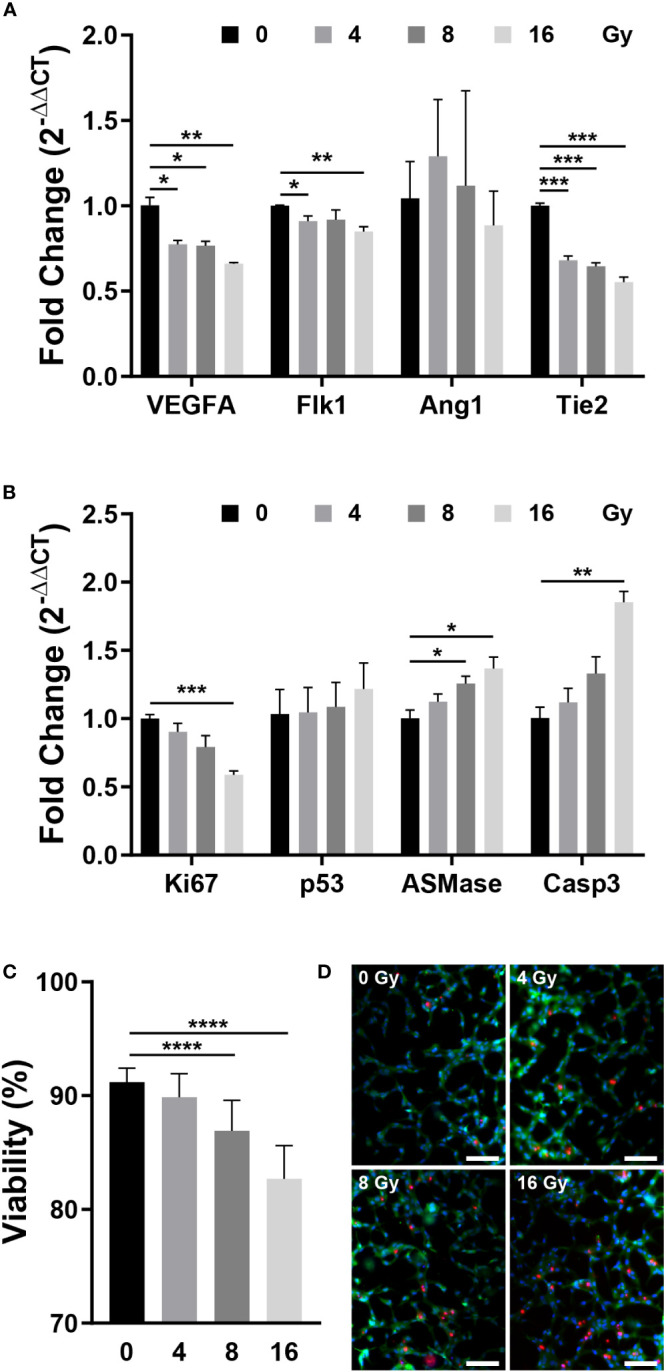
Gene expression and viability between control and irradiated vessels. **(A)** Gene expression of control and irradiated vessels. Flk1: VEGF receptor 2. Tie2: Ang1 receptor (mean ± SEM, n = 3). **(B)** Proliferative (Ki67) and apoptotic (p53, ASMase, Casp3) gene expression in control and irradiated vessels (mean ± SEM, n = 3). **(C)** Quantitative viability of control and irradiated vessels (mean ± standard deviation, n = 12). **(D)** Fluorescent images of vessel viability stained with the LIVE/DEAD kit. Scale bar, 100 μm. p53, TP53; ASMase, Acid sphingomyelinase; Casp3, Caspase 3. *p< 0.05, **p< 0.01, ***p< 0.001, ****p< 0.0001. SD, standard deviation SEM, standard error of the mean.

RT-qPCR was performed to measure the expression of genes that affect the vascular structure and those associated with apoptosis. VEGFA induces endothelial cell proliferation, inhibits apoptosis, increases vascular permeability, and promotes cell migration, and Flk1 is its receptor (2, 32, 36). Angiopoietin 1 (Ang1) contributes to the stabilization and protection of formed blood vessels, and Tie2 acts as its receptor (32, 35). After being exposed to radiation, the expression of VEGFA and Flk1 decreased substantially, while Ang1 showed no significant change and Tie2 expression decreased significantly (**Figure 3A**). Both the VEGFA-Flk1 and Ang1-Tie2 pathways decreased in the irradiated blood vessels. Ki67 and Caspase3 (Casp3) are expression markers of cell proliferation and apoptosis, respectively (37, 38). TP53 (p53) induces growth arrest or apoptosis in DNA-damaged cells, and ASMase mediates radiation-induced endothelial cell apoptosis (1, 7, 39). Following irradiation of endothelial cells, expression of Ki67 was decreased, whereas that of p53, ASMase, and Casp3 expression increased (**Figure 3B**). When exposed to radiation, blood vessels attempt to resist radiation damage and become unstable with decreased proliferation and increased apoptosis. To determine the effect of these phenomena on cells, the blood vessel viability was measured. Cell viability was measured using the LIVE/DEAD kit in the control group (0 Gy) and irradiated group, revealing that cells died in a dose-dependent manner (**Figures 3C, D**). These results suggested that radiation-exposed endothelial cells fail to form normal vascular networks owing to their instability, decreased proliferation, and increased apoptosis.

### Radiation induces the destruction of mature vascular networks

**Figure 4:**
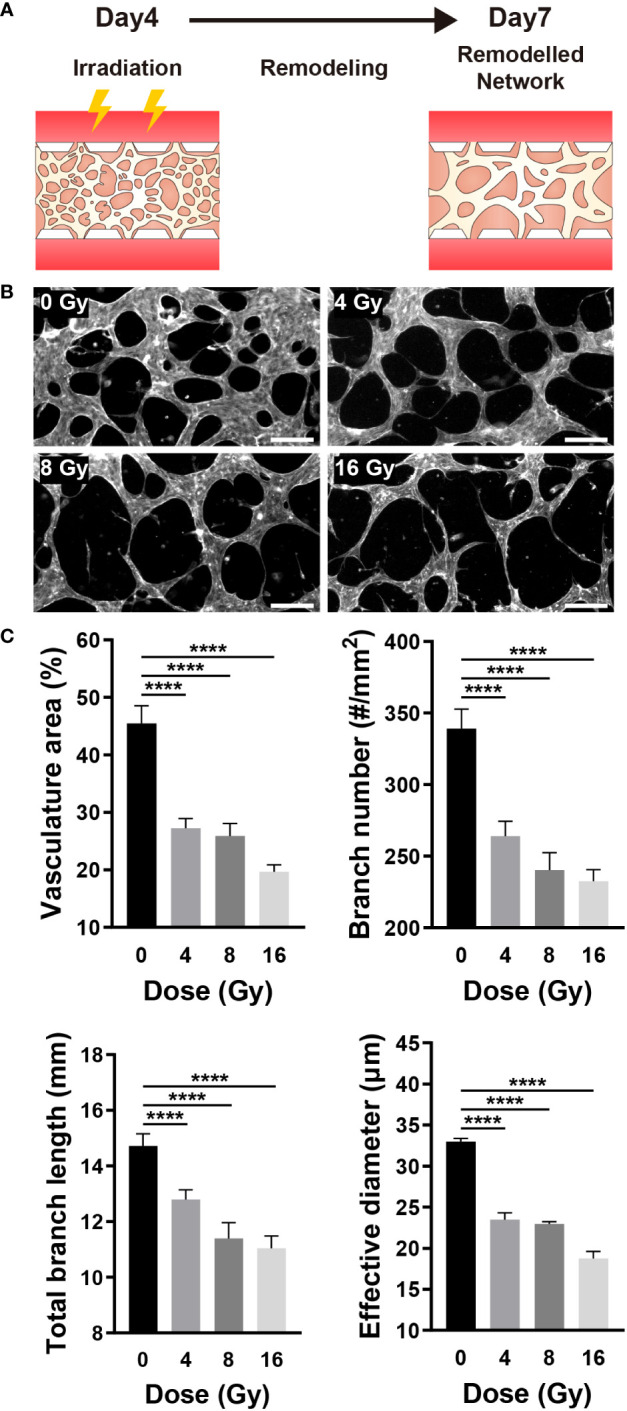
Radiation destroys the structure of the mature vascular network. **(A)** Schematic illustrating experimental conditions for analyzing the effect of radiation on the vascular network. **(B)** Control and irradiated vascular networks on day 7. Scale bars, 200 μm. **(C)** Quantitative indices such as vasculature area, number of branches, length of total branches, and effective diameter were measured to analyze the vascular structure of control and irradiated vessels (mean ± SD, n = 11). ****p< 0.0001. SD, standard deviation.

Given that blood vessels transport oxygen and nutrients to all parts of the body and remove waste products, the vascular network is essential for maintaining the homeostasis of tissues and cells (40). RT can affect the target, as well as surrounding vascular networks, thereby resulting in vascular dysfunction, inflammation, arteriosclerosis, and fibrosis (1). The blood vessel density, in other words, the oxygen saturation level, can substantially impact the RT results (1, 41). As previously stated, when a vascular network is formed from vascular progenitor cells, it is remodeled through branching, pruning, and intussusception. Similarly, on this platform, a vascular network was formed around day 4, followed by remodeling. To examine the effect of radiation on the vascular network, the network was established on a microfluidic chip and exposed to radiation, and changes in blood vessels were monitored (**Figure 4A**). Examining the remodeled network images, we confirmed that the blood vessels in the control group (0 Gy) formed a network structure, whereas the network structure was broken in the irradiated vessels (**Figure 4B**). To quantify changes in the morphology of the network, endothelial cells were fixed on day 7, and images of the entire network structure were measured using F-actin staining. Based on the staining images, the total area of the vasculature, the number of branches, the total length of the branches, and the effective vessel diameter were all measured using ImageJ. Based on quantitative assessments, we confirmed that the area of the vasculature, the number of branches, and the effective diameter of the 4 Gy and above irradiated group were significantly and dose-dependently decreased when compared with those of the control group; the 8 Gy and above irradiated group exhibited a significant reduction in the total branch length when compared with that of the control group (**Figure 4C**).

**Figure 5:**
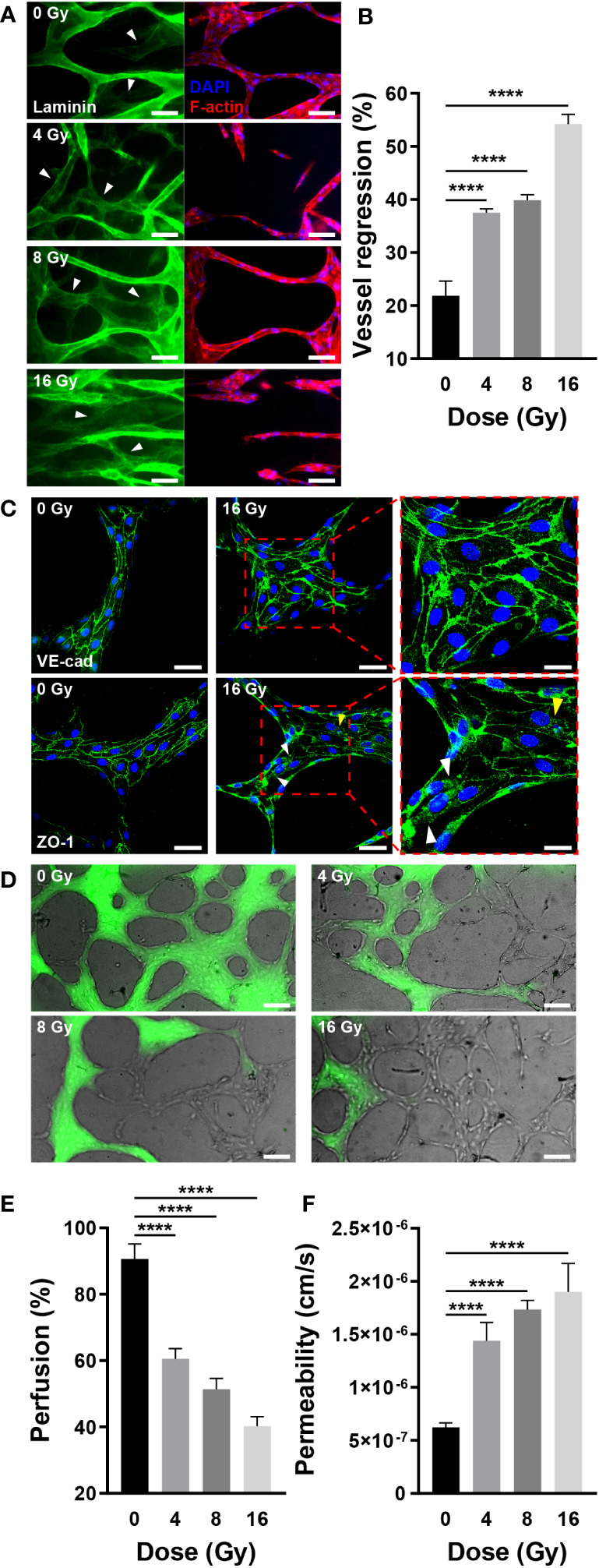
Radiation induces regression and dysfunction of the mature vascular network. **(A)** Fluorescent images of laminin (green, basement membrane), DAPI (blue, nuclei), and F-actin (red, F-actin) in control and irradiated vascular networks. White arrows indicate regions with laminin but without DAPI and F-actin. Scale bars, 50 μm. **(B)** Quantitative vessel regression figures are calculated by subtracting the area of F-actin from the area of laminin (mean ± SD, n = 7). **(C)** Junctions are destroyed when irradiated with 16 Gy. A radiation dose of 16 Gy damages tight junctions (ZO-1) but has little effect on adherens junctions (VE-cad). The white arrows indicate the locations of junctions destroyed by radiation, while the yellow arrow represents junction disruption induced by irradiated cell death. Scale bars, 50 (left & center) μm and 20 (right) μm. **(D)** Perfusion status of control and irradiated vessels with FITC-dextran. Phase contrast and FITC-dextran images were acquired concurrently. Scale bar, 100 μm. **(E)** Quantitative perfusion area is measured by dividing the dextran area by the vasculature area (mean ± SD, n = 13). **(F)** Permeability of control and irradiated vascular network (mean ± SD, n = 6). ****p< 0.0001. SD, standard deviation.

According to vascular network images and quantitative figures, the structure of radiation-exposed blood vessels was destroyed, decreasing the number of branches, diameter, and total length. This blood vessel regression was notable following laminin staining. As previously stated, the mature vascular network forms a basement membrane containing laminin, indicating that the laminin-stained sites are vascularized. When blood vessels were co-stained with laminin, F-actin, and nuclei, some vessels expressed laminin, F-actin, and nuclei, whereas others expressed only laminin (**Figure 5A**). Vessels stained exclusively with laminin regressed upon remodeling. For quantification, we measured the area excluding the F-actin-stained vascular area from the laminin-stained vascular area using ImageJ. Compared with the control group, irradiated vessels exhibited a significant and dose-dependent increase in the proportion of regressed blood vessels (**Figure 5B**). Similar to the results shown in **Figures 4B, C**, these results indicated that the vessels regressed in response to radiation exposure. Previously, we demonstrated how radiation destroys the structure of the vascular network, conducting experiments to investigate how radiation affects vascular functions, such as junction integrity, vascular blockage, and vascular permeability. Endothelial junctions, including adherens and tight junctions, and adhesion molecules are critical for intercellular communication, tissue integrity, and barrier function (42, 43). To check the junctional integrity of irradiated vessels, gene expression of vascular endothelial cadherin (VE-cad, adherens junction marker), zonular occludens-1 (ZO-1, tight junction marker), and intercellular adhesion molecule 1 (ICAM-1) was measured by RT-qPCR. The vessels were additionally stained and imaged with VE-cad and ZO-1 using confocal microscopy. After radiation exposure, VE-cad gene expression increased at 4 and 8 Gy, but returned to the control level at 16 Gy. However in fluorescent images, VE-cad expression in the control group was intact, and was not affected significantly by irradiation. ICAM-1 exhibited no significant changes in gene expression in response to irradiation, whereas ZO-1 exhibited a dose-dependent decrease in gene expression. It demonstrated a strong correlation with ZO-1 fluorescent staining images. Irradiation appeared to have severely disrupted and damaged the cell-to-cell tight junction (**Figures 5C**, **S2**, **S6A**). Blood vessels are critical for transporting blood containing oxygen and nutrients across the body and receiving and transporting carbon dioxide and waste products excreted by tissues and cells. Given that blood vessel blockage and permeability are critical for blood delivery through the vessels, we examined the effect of radiation on these functions. To confirm the blockage rate of the 3D vasculature, 70 kDa FITC-dextran was injected, and the amount of dextran flowing into the vasculature was measured after 30 s. Dextran was injected into the majority of the vasculature in the control group; however, a high proportion of vasculature did not receive dextran in the irradiated group (**Figure 5D**). As mentioned in the Methods section, the ratio of perfused vessel area was quantified by measuring the ratio of dextran-flowed vessel area to total vessel area. The ratio of perfused vessels decreased dose-dependently, and only ~40% of vessels perfused at 16 Gy (**Figure 5E**). To determine the mechanism underlying blood vessel occlusion, the blockage point was measured in three dimensions using confocal microscopy, confirming that the blood vessel, which appeared intact in the bright-field image, failed to function as a passage owing to the broken lumen (**Figure S3**). Permeability increased 2.31 times at 4 Gy, 2.78 times at 8 Gy, and 3.05 times at 16 Gy when compared to the control group (**Figures 5F**, **S4**). In order to corroborate endothelial dysfuction, the expression of endothelial nitric oxide synthase (eNOS) and von Willebrand factor (vWF) was also measured. Compared to the control group, eNOS levels in irradiated vascular networks decreased by 0.77-fold at 8 Gy and 0.75-fold at 16 Gy. Compared to the control group, the irradiation significantly increased the expression of vWF, with values of 1.38-fold at 4 Gy, 1.23-fold at 8 Gy, and 1.53-fold at 16 Gy (**Figure S6D**). Overall, these findings suggested that radiation disrupts vessel junctions, induces blockage by rupturing the lumen, and increases permeability, hindering the primary blood vessel functions of transporting and delivering substances into tissues and cells.

### Radiation induces DNA damage, apoptosis, and death in blood vessels

**Figure 6:**
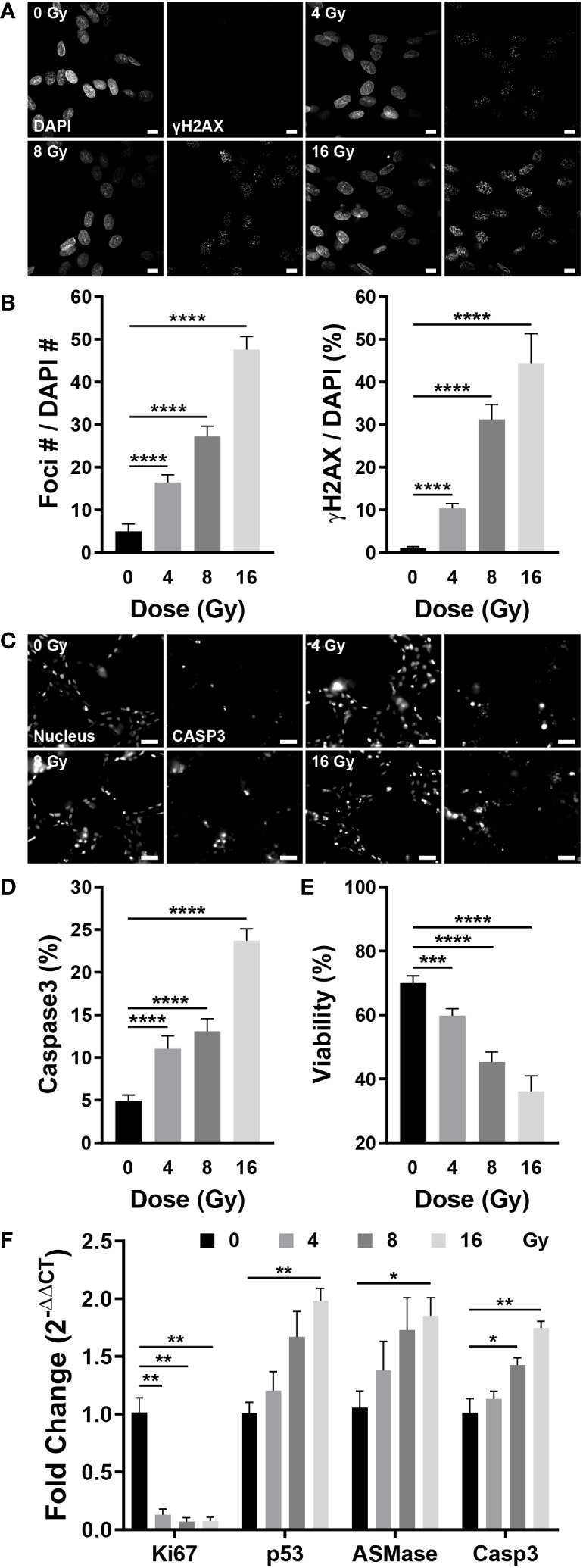
Radiation induces DNA double-strand breaks, apoptosis, and cell death in blood vessels. **(A)** Fluorescent images stained with DAPI and γH2AX, a double-strand break marker, in control and irradiated vessels. Scale bar, 10 μm. **(B)** The number of γH2AX foci and γH2AX area were quantified using the images in **(A)** (mean ± SD, n = 7). **(C)** Fluorescent images of Hoechst 33342 (nucleus) and NucView488 Caspase-3 in control and irradiated vessels. Scale bar, 50 μm. **(D)** Quantitative expression of caspase 3 is normalized by the nucleus (mean ± SD, n = 11). **(E)** Quantitative viability figures of control and irradiated vessels (mean ± SD, n = 9). **(F)** Proliferative (Ki67) and apoptotic (p53, ASMase, Casp3) gene expression in control and irradiated vessels (mean ± SEM, n = 3). *p< 0.05, **p< 0.01, ***p< 0.001, ****p< 0.001. SD, standard deviation. SEM, standard error of the mean.

Radiation damages the DNA of cells, either directly or indirectly. DSBs occur during this process, in which the double helix DNA structure is disrupted, which can be confirmed by γH2AX, produced by phosphorylation of H2AX, a variant of the H2A protein family (44). To examine whether radiation damages blood vessels, γH2AX was co-stained with DAPI to confirm DNA damage. Few γH2AX observed in the control group; however, irradiated vessels exhibited increased expression of γH2AX-specific foci (**Figure 6A**). Quantification of the number and area of foci based on the images revealed that the number of foci increased by 3.32 times at 4 Gy, 5.5 times at 8 Gy, and 9.61 times at 16 Gy when compared with that of the control group; the foci area increased by approximately 9.9 times at 4 Gy, 29.81 times at 8 Gy, and 42.39 times at 16 Gy when compared with that of the control group (**Figure 6B**). Given that cells with DSBs can either repair or undergo apoptosis, we examined apoptosis in blood vessels using NucView488 Caspase-3 and found that, similar to the DSB results, the incidence of apoptosis was increased in irradiated vessels (**Figure 6C**). Casp3 was quantified as a ratio to the nucleus in the region of interest, increasing by 2.25 times at 4 Gy and 4.81 times at 16 Gy when compared with that in the control group; the ratio increased in a dose-dependent manner (**Figure 6D**). The LIVE/DEAD kit was used to determine whether apoptosis resulted in blood vessel death. The viability decreased to 36.18% at 16 Gy when compared with 70% in the control group, decreasing in a dose-dependent manner (**Figure 6E**). Apoptosis was confirmed using gene expression analysis, and the results were similar to those obtained in experiments assessing the effects of radiation on neovascularization. The expression of apoptosis-related genes such as p53, ASMase, and Casp3 increased dose-dependently in response to irradiation, whereas the expression of Ki67, a marker of cell proliferation, was significantly decreased in the vascular network (**Figure 6F**). The expression of vascular-related genes revealed that VEGFA-Flk1 and Ang1-Tie2 were significantly decreased, consistent with the results observed in neovascularization. In addition, an increase in the expression of IL-6 confirmed that radiation induced an inflammatory milieu in the vasculature via an increase in inflammatory cytokines. (**Figure S6C**). Accordingly, radiation could induce DSBs in the vascular network, ultimately resulting in endothelial cell death and network destabilization.

### Radiation inhibits the blood vessel recovery

**Figure 7:**
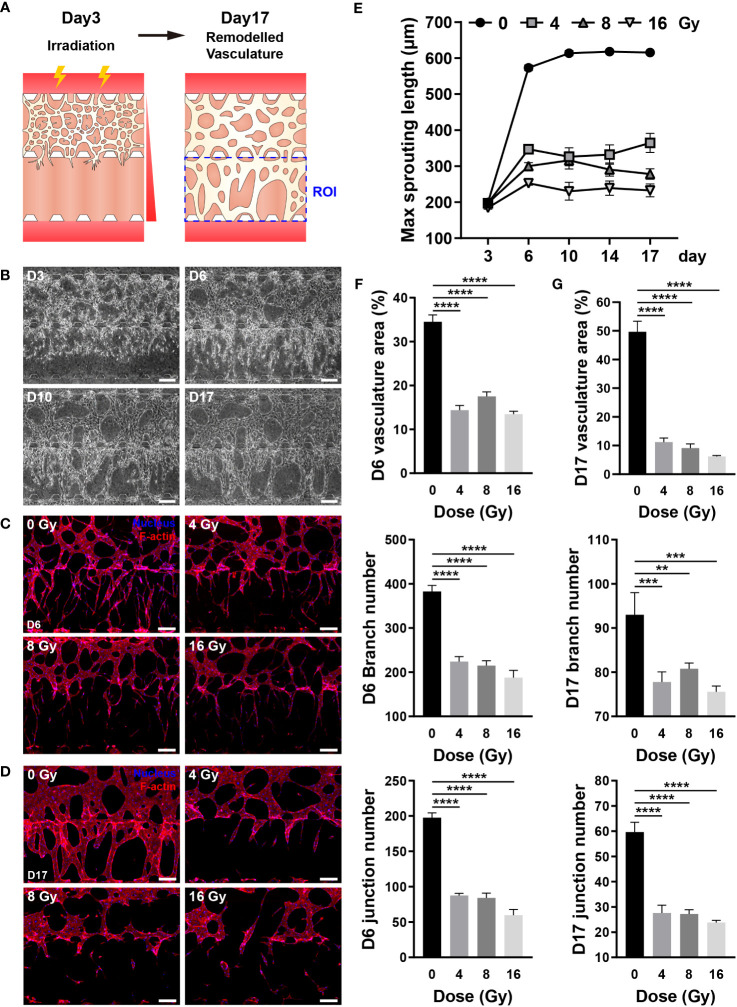
Radiation inhibits the recovery of three-dimensional (3D) vasculature. **(A)** Schematic illustrating experimental conditions for analyzing the effect of radiation on the recovery of the blood vessels. **(B)** Phase contrast images depicting the progression of vasculature morphology over time (Days 3, 6, 10, 17). Scale bars, 200 μm. **(C, D)** Fluorescent images of DAPI and F-actin in control and irradiated vessels at **(C)** day 6 and **(D)** day 17. Scale bar, 200 μm. **(E)** Quantification of the maximum sprouting length in control and irradiated vessels over time (mean ± SD, n = 5). **(F, G)** Vasculature area, number of branches, and number of junctions were measured to analyze the recovery of control and irradiated vessels using the images in **(C, D, F, G)** (mean ± SD, n = 8). **p< 0.01, ***p< 0.001, ****p< 0.0001. SD, standard deviation.

The blood vessel density can impact the concentration of oxygen, nutrients, hormones, and waste; hence, vessel recovery is crucial to mitigate the adverse effects of RT (45). Directionality was applied to blood vessel formation to easily and intuitively examine whether blood vessels recovered, and the chip structure was altered to provide directionality. As described in the Methods section, HUVECs were embedded in the upper fibrin gel, with no cells placed in the lower fibrin gel. A gradient of VEGFA and S1P was formed through media channels to guide vessel formation downward (**Figure 7A**) (46). In the control group, the vascular network was formed around day 3, blood vessels were formed at the end of the lower gel around day 6, followed by vessel formation and remodeling. Based on these results, the vessels were irradiated on day 3, and their recovery was confirmed for two weeks (**Figures 7A, B**). The recovery of irradiated vessels was confirmed by vessel formation in the lower gel channel. The irradiated vessels significantly differed from the control group vessels on day 6, with persistent damage observed even after two weeks (**Figure S5**). To analyze whether blood vessels had recovered quantitatively, the maximum sprouting length, which can be expressed most intuitively, was measured. On day 6, the maximum sprouting length of the irradiated group decreased from 0.441 to 0.605 times that of the control group, remaining nearly identical or decreased after two weeks (**Figure 7E**). To determine the vascular structure of each group in detail, the vessels were fixed on days 6 and 17, and the vasculature was quantified by nuclear and F-actin staining. On day 6, control vessels had a long sprouting length and numerous branches, and the branches were entangled to form a complex structure. Conversely, irradiated vessels exhibited a short sprouting length, a significantly reduced number of branches, with a simple structure (**Figure 7C**). More precisely, the control vessels occupied a 34.52% area ratio and comprised 382.83 branches and 197.67 junctions, whereas the irradiated vessels occupied a 13.48–17.51% area ratio and comprised 187.75–224.25 branches and 59.75-87.5 junctions (**Figure 7F**). On day 17, the control vessel area and thickness were increased, and the structure was simplified when compared with that on day 6. Likewise, the thickness of the irradiated vessel was increased, and the structure was simplified; however, the area was decreased when compared with that on day 6 (**Figure 7D**). According to quantitative analysis, the area ratio of vessels in the control group was 49.68%, with 93 branches and 59.6 junctions, whereas the irradiated vessels accounted for 6.24–11.21% of the area, with 75.6–80.8 branches and 23.8–27.6 junctions (**Figure 7G**). The results of gene expression analysis of hypoxia-inducible factor 1-alpha (HIF-1a), VEGFA, integrin alpha-v (ITGAV), and integrin beta-3 (ITGB3) implicated in vascular recovery in irradiated blood vessels revealed that HIF-1a did not exhibit irradiation-related differences. VEGFA decreased substantially at 4 Gy and remained below 50% of the control group at doses of 8 Gy or higher. At 4 and 8 Gy, there were no significant differences in ITGAV, but at 16 Gy, there was a significant decrease. ITGB3 showed a significant increase at 4 and 8 Gy, but no significant difference at 16 Gy (**Figures S6B, C**). Based on these findings, it was confirmed that blood vessels damaged by radiation doses exceeding 4 Gy lose their ability to vascularize; hence, the vessels cannot be recovered, with further structural deterioration.

## Discussion

RT, along with surgery and chemotherapy, is a common strategy to treat cancer and provide palliative care. Radiation kills cells by damaging their DNA structure, affecting both normal and malignant cells. To reduce damage to normal cells and enhance cancer cell damage, radiation is fractionated, and technologies such as LET, SRS, and SBRT have been developed to improve the efficacy of RT, although short- and long-term toxicities persist (8–10, 47). Numerous radiosensitive capillaries are distributed throughout the brain, heart, lungs, kidneys, and digestive tract, well-known organs that exhibit irradiation-induced tissue damage. Irradiated capillaries cause alveolar edema, exudation, and vascular congestion in the lungs, blood-brain barrier disruption in the brain, and pericarditis in the heart (18, 48–50). Endothelial cell apoptosis can cause gastrointestinal damage (51). Microvasculature-on-a-chip, a 3D model of pathophysiological microvessel networks, can form 3D capillaries in response to chemical stimuli. This model demonstrated RT-induced acute and subacute phenomena in human capillaries. Microvasculature-on-a-chip, a 3D model of pathophysiological microvessel networks, can form 3D capillaries in response to chemical stimuli. This model demonstrated RT-induced acute and subacute phenomena in human capillaries. At day 7, the average diameter of microvessels formed in the chip was 36.8 µm, which was larger than capillaries (4~8 μm) and within the average range of blood microvessels (5~70 μm) (52, 53).

Radiation-induced vascular injury includes DNA damage, senescence, and death from the perspective of endothelial cells; and oxidative stress, inflammatory state and fibrosis as a result of elevated ROS, inflammatory cytokines, and transforming growth factor (TGF)-β from the perspective of the vascular milieu. Considering a vascular network structure, radiation-mediated vascular injury encompasses several manifestations such as reduced blood vessel density, stenosis, coagulation, disruption of barrier homeostasis, enhanced permeability, and endothelial cell detachment from the basement membrane (1, 16, 48, 54, 55). In the vasculature-on-a-chip, irradiated endothelial cells embedded in the ECM exhibited DNA damage, decreased proliferation and increased apoptosis, resulting in a reduced survival rate. The most significant advantage of the microfluidic format may be the ability to effectively monitor structural morphogenesis in 3D. Neighboring stromal tissues and ECMs needs to be integrated in the future study, for complete verification of the complicated vascular milieu. Accordingly, the total area, length, and the number of branches and nodes of the network were reduced. On exposing network-forming blood vessels to radiation, DNA damage accumulated in the endothelial cells, apoptosis increased, and the survival rate decreased. Consequently, the overall area, length, number of branches, and branch diameter of the network decreased. Vascular degeneration, previously observed *in vivo* only, was confirmed in the present model, as determined by the expression of laminin, F-actin, and DAPI, with vascular regression increasing in proportion to the radiation dose (56). Changes in junction integrity, occlusion, and permeability were confirmed to verify that irradiation could alter blood flow, a fundamental function of blood vessels. Adherens junctions exhibited no significant differences in response to radiation exposures up to 16 Gy, whereas tight junctions demonstrated a dose-dependent decrease in mRNA levels. However, only at 16 Gy morphological disruption was observed in fluorescent images. This suggests that tight junctions rather than adherens junctions play a more dominant role in the permanent reduction of blood flow in response to strong irradiation exposure (1). Interestingly, two phenomena were identified in the breakdown of junctions: breakdown of the junction itself and breakdown of the junction caused by cell death (**Figure 5C**). The junctions exhibited significant differences on exposure to 16 Gy, whereas vascular occlusion and permeability showed significant functional differences at 4 Gy. In addition to the decreased function due to reduced blood vessel density, the decreased perfusion caused by blood vessel occlusion increased proportionally with the dose of irradiation, and did the permeability. eNOS is a key regulator in the maintenance of endothelial homeostasis, which includes endothelial membrane function, the coagulation cascade, membrane permeability, and membrane integrity. It generates the vasoprotective molecule nitric oxide (NO), which promotes the health of blood vessels. Reduced expression of eNOS has been linked to endothelial dysfunction (57, 58). When endothelial cells are damaged, vWF expression increases, and this increase is associated with atherosclerosis and thrombosis (59). Experimentaly observed diseases in eNOS expression and increases in vWF expression provide additional evidence that radiation disrupts vascular function. Stroke and myocardial infarction can be caused by structurally and functionally degenerated vascular network; therefore, it is crucial to determine whether radiation-damaged blood vessels can recover (48). Therapeutic angiogenesis aims to restore normal blood flow to ischemic tissues by inducing the formation of new vascular networks with the administration of specific growth factors. Promoting proangiogenic pathways, such as VEGFA, a key regulator of vascular growth and therapeutic angiogenesis, increases vascular density and perfusion, offering therapeutic potential against diseases characterized by impaired blood flow, including peripheral artery disease, ischemic heart disease, and ischemic stroke (45, 60, 61). Angiogenesis was induced by establishing a gradient of combination of VEGFA and S1P on the chip to determine whether irradiated blood vessels with deteriorated structure and function could be regenerated. ITGAV, ITGB3, HIF-1a, and VEGFA are involved in vascular system recovery. The expression of integrins implicated in endothelial migration and tube formation, specifically ITGAV and ITGB3, was altered in irradiated vascular networks (62). ITGAV expression decreased marginally at 16 Gy, while ITGB3 expression increased slightly at 4 and 8 Gy. The effect of irradiation on the expression of HIF-1a was not statistically significant. VEGFA operates as a downstream angiogenic mediator of HIF-1a, promoting angiogenic functions such as endothelial function, migration, survival, and facilitating endothelial recovery (63–65). The expression of VEGFA decreased by approximately 40% at 4 Gy and by more than 60% at dosages greater than 8 Gy. A decrease in nitric oxide synthase expression inhibits the function and regenerative capacity of endothelial cells (57). Therefore, the decrease in VEGFA and eNOS expression in the irradiated vascular network played a crucial role in inhibiting vascular regeneration. The radiation-exposed blood vessels exhibited approximately half the sprouting ability of the control group on day 3 post-exposure and failed to grow from day 3 onward until day 14. Based on values of structural indicators on days 3 and 14 of radiation exposure, blood vessels failed to recover from radiation damage, even after 14 days. It is hypothesized that to form new blood vessels, irradiated vessels require a supply of vascular progenitor cells or other factors that aid in the formation of new blood vessels. Using microvasculature-on-a-chip, irradiated blood vessels exhibited *in vivo*-reported phenomena and quantitatively demonstrated vascular structural degeneration, functional decline, and suppressed regeneration following irradiation.

Herein, we constructed a 3D microvasculature model and quantitatively analyzed the radiation-induced adverse effects on blood vessels. Using human dermal microvascular endothelial cells in previous *in vitro* models, the adverse effects of radiation on endothelial monolayers were confirmed. Similar results for adherens junctions, tight junctions, and permeability indicated that our 3D microvasculature could mimic microvessels (66). Using the 3D microvasculature model, it was also possible to observe adverse effects of radiation on the vasculature at its maturation status. The adverse effects of radiation exposure on blood vessels were analyzed from the perspectives of endothelial cells, endothelial cell layer, and vascular network. Radiation interferes with neovascularization, induces apoptosis, damages vascular structures, deteriorates blood flow transport function, and results in negligible recovery from radiation-induced damage. On the basis of the unique characteristics of microvasculature-on-a-chip, including physiological structure, chemical gradients, and high-resolution imaging, we have developed an efficient model and analysis method for radiation-induced acute and subacute vascular regression. However, one difficulty we encountered was the difficulty of protein analysis due to chip’s scale. It is very challenging, and need to be explored further. This model is suitable for analyzing the response of blood vessels to radiation therapy for bone marrow or cancer, as well as the promotion or prevention of vascular injury.

## Data availability statement

The original contributions presented in the study are included in the article/**Supplementary Material**. Further inquiries can be directed to the corresponding authors.

## Ethics statement

Ethical approval was not required for the studies on humans in accordance with the local legislation and institutional requirements because only commercially available established cell lines were used.

## Author contributions

D-HC designed and performed the experiments and wrote the manuscript. DO, KN, and HK contributed to conducting experiments and analyzing results. YJ, JA, and JK provided advice for designing experiments. DC and C-HK assisted with the irradiation process. SC supervised the research, authored the manuscript, and provided financial support. All authors contributed to the article and approved the submitted version.
