# Supplementary material for: Radiation induces acute and subacute vascular regression in a three-dimensional microvasculature model
Source: Front Oncol. 2023 Oct 16;13:1252014. doi: 10.3389/fonc.2023.1252014 (PMC10613678; doi:10.3389/fonc.2023.1252014)
Supplement: Supplementary file 1 [file DataSheet_1.docx]

Supplementary Material

Radiation induces acute and subacute vascular regression in a three-dimensional microvasculature model

Dong-Hee Choi^1,5^, Dongwoo Oh^3^, Kyuhwan Na^1,5^, Hyunho Kim^1,6^, Dongjin Choi^2^, Yong Hun Jung^1,5^, Jinchul Ahn^1,5^, Jaehoon Kim^1,7^, Chun-Ho Kim^2*^ and Seok Chung^1,3,4*^

*** Correspondence:** Seok Chung: [sidchung@korea.ac.kr](mailto:sidchung@korea.ac.kr), Chun-Ho Kim: [chkim@kirams.re.kr](mailto:chkim@kirams.re.kr)


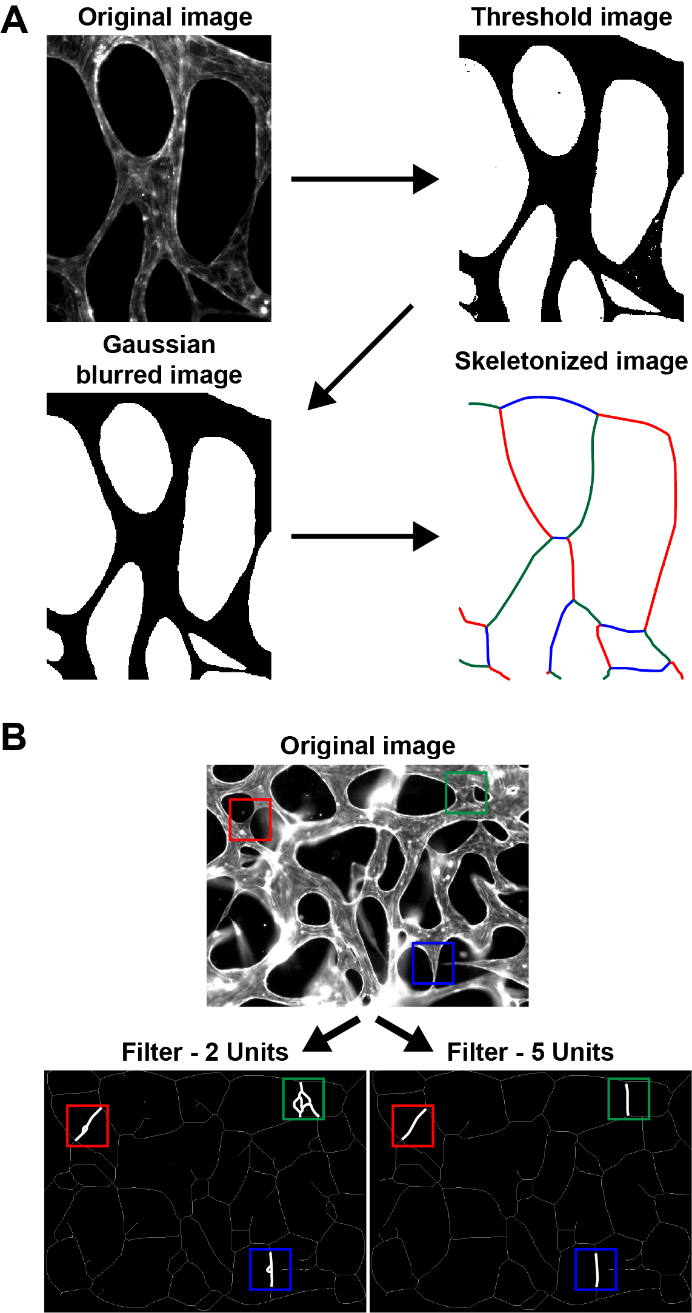


**Fig. S1. The process of analyzing the structure of the vasculature. (A)** Threshold and Gaussian blur were processed using fluorescent images with ImageJ. The vasculature was analyzed using Analyze Skeleton (2D/3D). **(B)** Skeletonized images after 2-unit and 5-unit Gaussian blur filter applied.


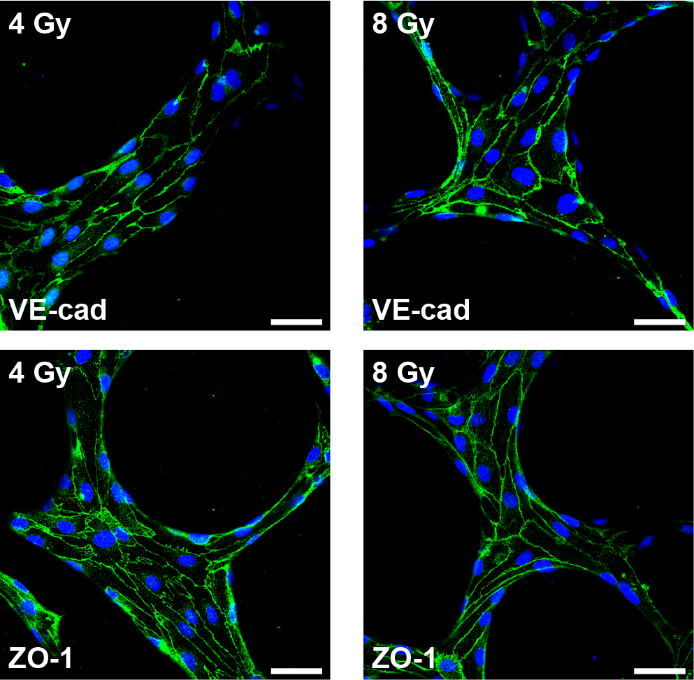


**Fig. S2. Junction integrity of exposed vasculature at 4 and 8 Gy.** Adherens (VE-cadherin) and tight (ZO-1) junction states of vessels exposed to 4 and 8 Gy doses of radiation. Scale bar, 50 μm.


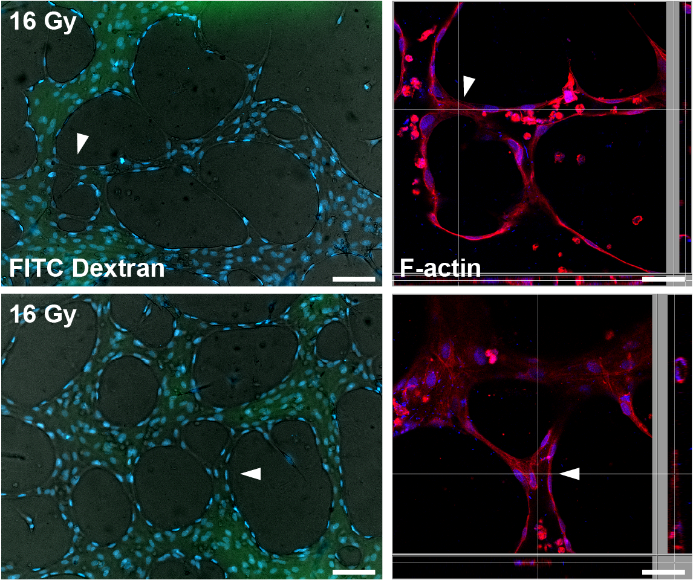


**Fig. S3. The blockage of vessels irradiated with 16 Gy.** The perfusion status of vessels exposed to 16 Gy irradiation was measured using 70 kDa FITC-dextran. The vessels were stained with Hoechst 33342 and F-actin, and the lumen structure was confirmed by confocal microscopy. The white arrows indicate the locations where the lumen structure was broken. Scale bar, 100 (Dextran) and 50 (F-actin) μm.


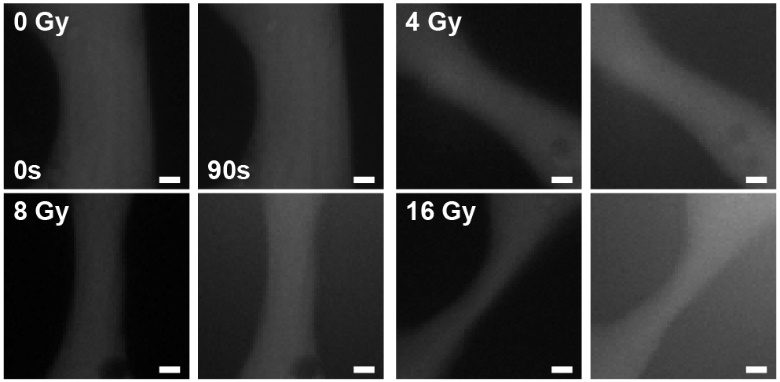


**Fig. S4. Blood vessel permeability in the control and irradiated states.** Vascular permeability was measured using 70 kDa FITC-dextran, and images were obtained every 10 s following dextran perfusion. Scale bar, 10 μm.

**
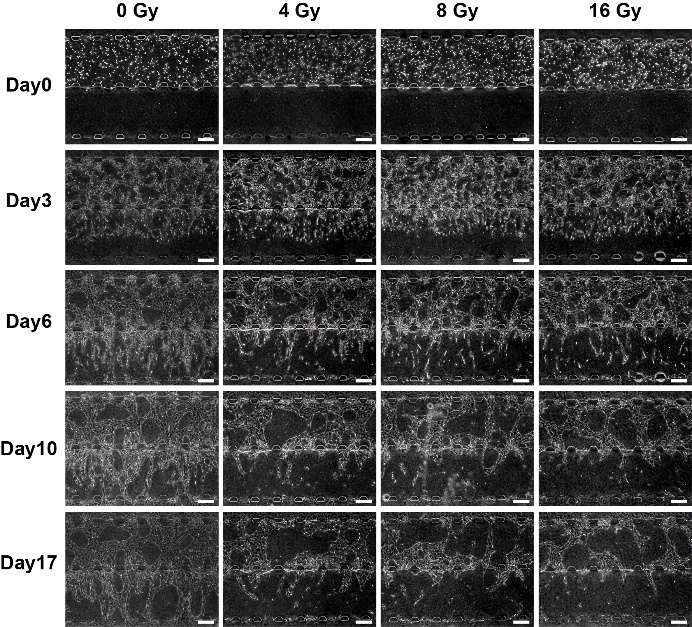
**

**Fig. S5. The recovery status of control and irradiated vessels.** Phase contrast images illustrate the progression of the morphology of control and irradiated vasculature over time (Days 0, 3, 6, 10, 17). Scale bar, 200 μm.


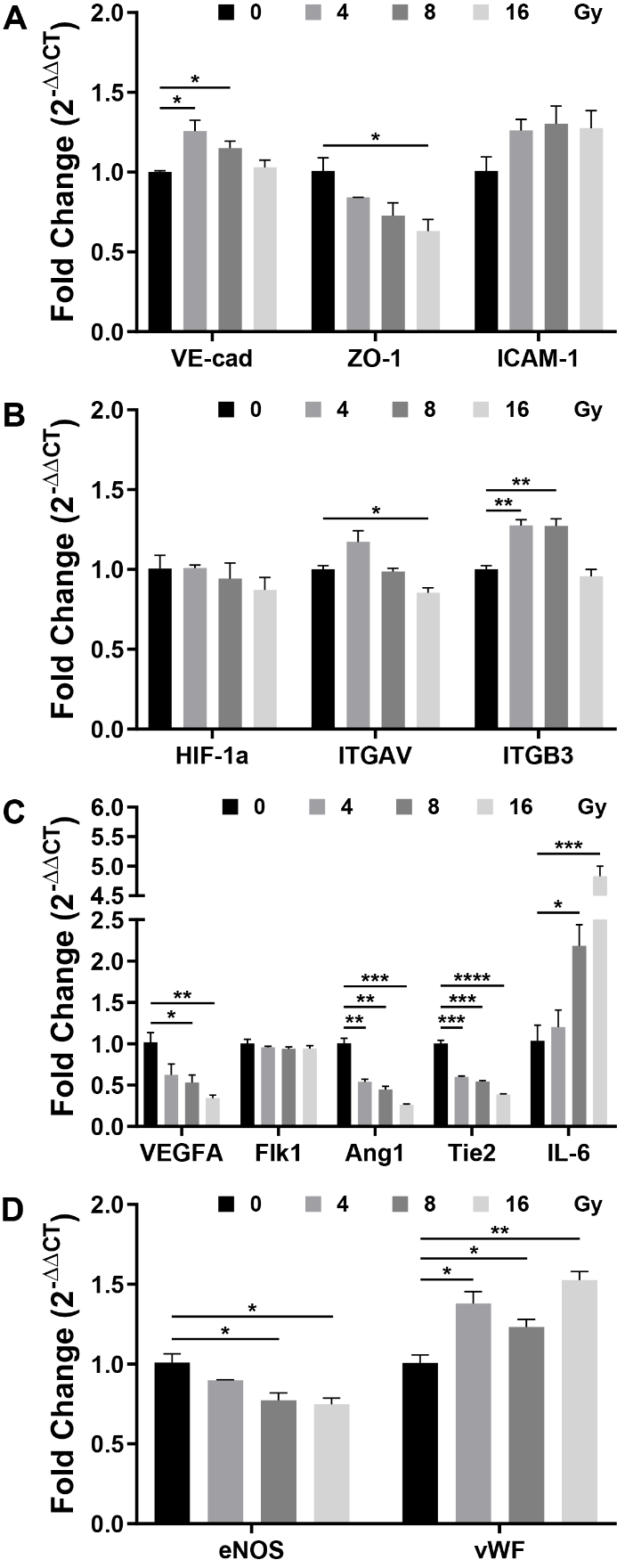


**Fig. S6. Vascular gene expression in control and irradiated vascular networks. (A)** Gene expression levels of junctions and adhesion molecule were measured in control and irradiated vascular networks using RT-qPCR (mean ± SEM, n = 3). **(B)** Expression of vascular recovery markers (mean ± SEM, n = 3). **(C)** Expression of vascular markers and inflammatory markers (mean ± SEM, n = 3). **(D)** Expression of endothelial functional markers (mean ± SEM, n = 3). **p* < 0.05, ***p* < 0.01, ****p* < 0.001, *****p* < 0.0001. RT-qPCR, reverse transcription-quantitative PCR; SEM, standard error of the mean.

**Table S1. Primer sequences for RT-qPCR.**

| VEGFA | GGAAAGGGGCAAAAACGAAA |
| --- | --- |
|  | TGCAACGCGAGTCTGTGTTT |
| Flk1 | agtctgtggcatctgaaggc |
|  | ccgagtcaggctggagaatc |
| Ang1 | Gcctgatcttacacggtgct |
|  | Cgtaaggagtaactgggccc |
| Tie2 | TTGAAGTGGAGAGAAGGTCTG |
|  | GTTGACTCTAGCTCGGACCAC |
| Ki67 | tcgaccctacagagtgctca |
|  | gtggggagcagaggttcttc |
| p53 | TGGCCATCTACAAGCAGTCACA |
|  | GCAAATTTCCTTCCACTCGGAT |
| ASMase | TGGCTCTATGAAGCGATGGC |
|  | TTGAGAGAGATGAGGCGGAGAC |
| Casp3 | aaataccagtggaggccgac |
|  | aacccgggtaagaatgtgca |
| GAPDH | gatttggtcgtattgggcgc |
|  | ttcccgttctcagccttgac |
| HIF-1a | GCGCGAACGACAAGAAAAAG |
|  | TGTGGAAGTGGCAACTGATGA |
| ITGAV | GGAGCAATTCGACGAGCACT |
|  | GATTCATCCCGCAGATACGC |
| ITGB3 | AAGTGCGTGACCTCCCTGAA |
|  | ACCTTGGCCTCAATGCTGAA |
| VE-cad | GGCTGGACCGGGAGAATATC |
|  | GAACAACCGATGCGTGAACA |
| ZO-1 | CACTGGTGAAATCCCGGAAA |
|  | GCCATCTCTTGCTGCCAAAC |
| ICAM-1 | CTCCCCCACAACTTGTCAGC |
|  | CTGTGGGGTTCAACCTCTGG |
| eNOS | CTGAGATCGGCACGAGGAAC |
|  | CTTTCCACAGGGACGAGGTG |
| vWF | ACACCTGCATTTGCCGAAAC |
|  | TGGCAGATCCCACTGAAGGT |
| IL-6 | tgcaataaccacccctgacc |
|  | atttgccgaagagccctcag |
